# Digital: accelerating the pathway

**DOI:** 10.1098/rsta.2023.0411

**Published:** 2024-10-09

**Authors:** Andrew Davis, Chris Waldon, Stuart I. Muldrew, Bhavin S. Patel, Patricia Verrier, Thomas R. Barrett, Gerasimos A. Politis

**Affiliations:** ^1^ United Kingdom Atomic Energy Authority, Culham Campus, Abingdon, Oxfordshire OX14 3DB, UK

**Keywords:** step, design, modelling, multi-scale, extrapolation, digital twin

## Abstract

The Spherical Tokamak for Energy Production (STEP) programme is an ambitious but challenging endeavour to design and deliver a prototype fusion power plant. It is a rapid, fast-moving programme, designing a first of a kind device in a Volatile, Uncertain, Complex and Ambiguous (VUCA) environment, and digital tools play a pivotal role in managing and navigating this space. Digital helps manage the complexity and sheer volume of information. Advanced modelling and simulation techniques provide a platform for designers to explore various scenarios and iteratively refine designs, providing insights into the intricate interplay of requirements, constraints and design factors across physics, technology and engineering domains and aiding informed decision-making amidst uncertainties. It also provides a means of building confidence in the new scientific, technological and engineering solutions, given that a full-scale-integrated precursor test is not feasible, almost by definition. The digital strategy for STEP is built around a vision of a digital twin of the whole plant. This will evolve from the current digital shadow formed by system architecting codes and complex workflows and will be underpinned by developing capabilities in plasma, materials and engineering simulation, data management, advanced control, industrial cybersecurity, regulation, digital technologies and related digital disciplines. These capabilities will help address the key challenges of managing the complexity and quantity of information, improving the reliability and robustness of the current digital shadow and developing an understanding of its validity and performance.

This article is part of the theme issue ‘Delivering Fusion Energy – The Spherical Tokamak for Energy Production (STEP)’.

## 1. STEP digital vision

**Figure 1.**
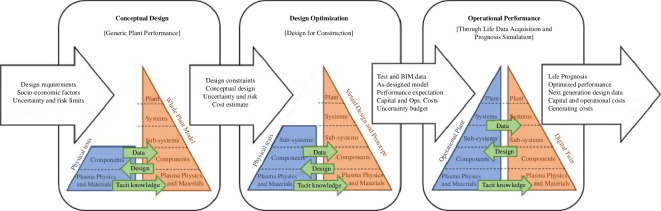
Schematic diagram of the evolving interface between the real-world (blue) and virtual environment (orange) (Reproduced from [1]). Note BIM stands for Building Information Modelling.

At the heart of the Spherical Tokamak for Energy Production (STEP) programme’s digital strategy is a vision to reduce the technical risk and, in the best-case scenario, accelerate delivery via the development of a digital twin for a first power plant class device such as STEP [1]. A digital twin is a virtual representation of the real-world machine linked to real-time observations, giving an identical replica of performance and control. By creating a digital twin, the team can optimize the design, operation and maintenance of both the STEP prototype power plant and its commercial successors, as well as substantially reduce the uncertainties and costs associated with physical testing (see figure 1). A digital twin is adaptable and scalable, with its effectiveness shaped by how it is tailored to specific use cases. The context of its application influences how we construct the model and evaluate its performance [2,3].

To achieve this vision, a digital shadow first needs to be built. The digital shadow is not a full replication like the digital twin, however, it gives the overall behaviour. This is a preliminary numerical model of the fusion device that enables discovery and innovation. It allows the design team to hypothesize and test new ideas through computational methods that augment human creativity. The digital shadow will incorporate data and knowledge from multiple sources, such as physics-based simulations, data-based surrogate models and experimental observations and, as shown in figure 1, will evolve from conceptual design to design optimization.

**Figure 2.**
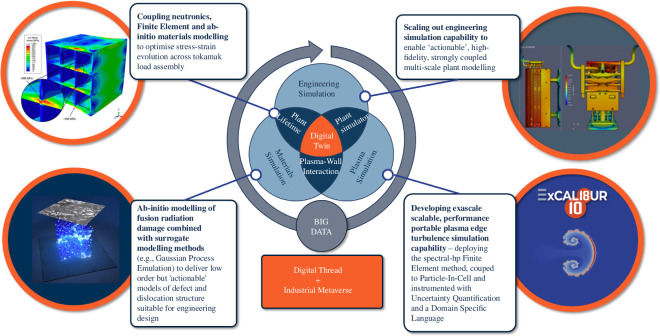
Building towards a digital twin.

The true value of the digital shadow then lies in its transition to the digital twin. This transition will be enabled by careful physical testing and validation of the digital shadow’s predictions. Through this process, the design team will gain a holistic understanding of the STEP device, from the atomic to the system level (see figure 2). The digital twin will then be able to learn from the real-world data and feedback, providing reliable and actionable insights. Beyond STEP, this should reduce the time and cost of developing and deploying new improved fusion power plant designs that can meet the diverse and changing needs of the energy market.

The modelling and simulation of systems or, eventually, a whole plant to form a digital twin necessitates a framework that includes a series of interconnected, multi-scale, multi-physics models linked to real-world data through the complete lifecycle. The UK Government recognized the need for such an integrated approach in the domestic nuclear sector. As a result, through the Department of Business, Energy and Industrial Strategy (BEIS), now the Department for Energy Security and Net Zero (DESNZ), it funded the Nuclear Virtual Engineering Capability Project (NVEC) (previously known as Digital Reactor Design) as a driver for further work on the Integrated Nuclear Design Environment (INDE) and a proof of principle high-level architecture [4]. Whole plant simulation in tokamak design offers a transformative opportunity to integrate advanced computational techniques with data-driven insights and contextual understanding.

This vision is ambitious but achievable and will require significant investment in the best hardware, software and talent, as well as close collaboration with strategic partners and a wide range of stakeholders to ensure alignment and integration across a wide range of digital themes.

## 2. The STEP digital shadow

The eventual foundational model for the digital twin as in figure 1 is a model-based systems engineering (MBSE) schema (see [5]). During the current conceptual phase, this is captured through system architecting parametric tools, which act as a consistency check at the whole plant level. These tools alongside occasional high-fidelity modelling form the current STEP device digital shadow. This shadow informs design and concept selection, so must be both flexible and fast, while reflecting the level of detail in the concepts.

As discussed in [6], the STEP device is a complex system with high degrees of connectivity, and interdependencies between systems become clearer with every design iteration. This interconnectivity drives the design process, as shown in fig. 1 in [5], and hence the current logical flow in the digital shadow: it is not a direct time-stepping simulation of the entire plant, but instead scenario modelling using a linked series of workflows focusing on specific systems and subsystems to analyse performance and assist in refining each concept. The details of this approach for the start of this overall workflow—the plasma design—are illustrated in §2*b*, while some of the more downstream components—the in-vessel systems—are described in §2*b*. Similar interconnected workflows exist for other systems—for example, the integrated design tools used to provide quick estimates of the minimal physical sizes of each magnet system as discussed in [7]; the systems modelling approach to the tritium fuel cycle design described in [8], and the approach to designing the control systems discussed in [9].

### (a) Digital plasma design

Advances in plasma simulation will ultimately lead to the development of rapid, high-fidelity integrated models; however, in the interim, we need to progress the design while developing these tools. The current approach taken on STEP has been to develop workflows, by linking low-fidelity codes, to create concepts. These concepts can then be used as the input to slower, higher fidelity modelling [10]. At the centre of the device is the plasma, which sets several requirements for the engineering of the machine, thus it is paramount that the initial design work focuses on the plasma–machine interaction.

**Figure 3.**
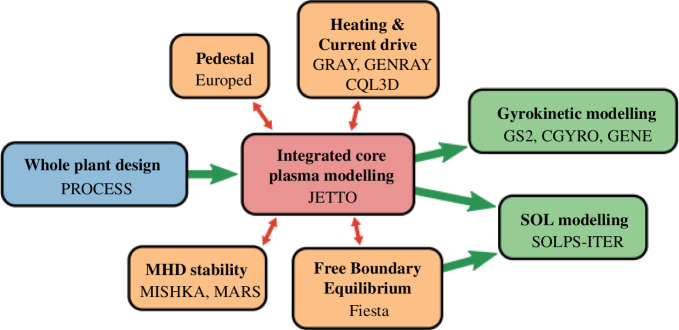
The STEP workflow for plasma flat-top design. (Reproduced from [11]).

Figure 3 illustrates the workflow used to develop a plasma scenario and demonstrates this progression from low- to high-fidelity modelling [11]. It begins with the open-source systems code PROCESS [12–14] to develop an initial plant design. PROCESS contains simplified models for the entire fusion powerplant: from plasma through to machine design, the power balance giving the net electric output and a cost model. It is also coupled to an optimizer allowing it to solve for a given figure-of-merit, such as the smallest machine that will give 100 MW_e_ of net electrical output. Based on a set of input assumptions and constraints, PROCESS will give the major plant parameters, such as fusion power and size. It achieves this by developing the requirements of the plasma, linking to the machine through the heat loads and toroidal magnetic field strength required. Using current densities and stress limits, it can size the magnets which in turn contribute to sizing the machine. The advantage of PROCESS is it can rapidly iterate through many combinations until it finds a design that meets the constraints. All of this can be completed within around 10 s on a single-core compute node.

Once this starting point has been developed, the size, toroidal field strength, fusion power and fusion gain requirements can be fed into the 1.5-dimensional-integrated core plasma code JETTO [15], allowing the solution to be verified and further developed. At this stage, PROCESS and JETTO are being run as ‘assumption integrators’, i.e. they are being used to do analysis based on assumed behaviour rather than first-principle calculations. It is then the need for higher fidelity codes to verify the assumptions that have been made. Additional codes can then be run to feedback into the JETTO solution, shown in orange in figure 3. These address some of the key challenges around the plasma design such as assessing plasma stability, heating and current drive requirements and positioning the poloidal field coils. The exact set-up of JETTO can vary depending on how many additional models are run alongside it but runs typically range from core-minutes for the simplest to core-weeks for the more detailed runs. Furthermore, the integration of physics and technology is made possible by using frameworks, such as BLUEMIRA [16,17]. The speed of PROCESS and JETTO makes them excellent tools for parameter space exploration and uncertainty quantification. This allows for the creation of reliable inputs for the high-fidelity codes, such as gyrokinetics and scrape-off layer modelling, which take a significant amount of time.

Turbulent transport is expected to play a significant role in the core performance of STEP plasmas. A detailed description of the STEP plasma is given in Meyer *et al*. [18], however we will only cover the modelling aspects here. STEP will operate in a regime far away from the currently explored operating space meaning current confinement scaling laws cannot be used with great confidence. To understand the expected level of transport, a fundamental knowledge of the turbulent characteristics in a high *β* (ratio of plasma-to-magnetic pressure) spherical tokamak regime is required. The most sophisticated turbulence model currently being employed is gyrokinetics, though the use of a gyrokinetic solver is computationally intensive, especially given the high *β* electromagnetic nature of the turbulence generated in the STEP regime.

The dominant linear local instabilities in the core plasma at a STEP flat-top operating point have been identified as hybrid kinetic ballooning modes (KBMs) and microtearing modes (MTM), which are both highly electromagnetic in nature [19]. The nonlinear space has been explored extensively where the hybrid KBMs were found to be the dominant source of transport and the dependencies on plasma parameters have been characterized. Significant effort has been put into running the simulations to turbulence saturation which is especially difficult in electromagnetic regimes [20,21]. This work has motivated the use of nonlinear global simulations in certain regimes of the operating point which is currently being explored.

Obtaining a single saturated, converged plasma turbulence simulation can require millions of core hours. Exploring the parameter space to optimize the core plasma via integrated modelling that embeds such simulations is not currently feasible, which has led to the development of reduced transport models applicable in this regime. An example is the quasilinear transport model, which has been developed using much more computationally efficient linear gyrokinetic simulations along with a saturation model developed from a range of STEP- relevant nonlinear simulations. With this model, flux-driven calculations have been performed for the flat-top operating point where the predicted turbulent fluxes were found to be compatible with the available heating sources [22].

Modelling of the plasma current ramp-up phase has been completed using both linear and nonlinear gyrokinetics using the code CGYRO [23]. This ramp-up scenario was modelled using JINTRAC [24] with a simple Bohm–gyroBohm model for the turbulent transport. This work then looked at various times along the ramp-up to determine what transport would be generated using this higher fidelity gyrokinetic model. Simulations were performed examining a core flux surface of ψ_Ν_ = 0.5 at several times ranging from 100 up to 2500 s at which point the plasma had reached the flat-top operating point. A transition point from electrostatic to electromagnetic turbulence was identified as well as a stabilization of modes at the electron Larmor radius scale, encouraging the use of ion scale simulations. The early phase at lower *β* was found to saturate with electrostatic turbulence while the later higher *β* plasmas were found to saturate with electromagnetic turbulence. An intermediate regime was found where the fluxes increased significantly as the increased drive from higher *β* was not being compensated by the increased stabilization from radial gradients in *β*, *β*′. The substantial increase in fluxes was found to disappear when parallel magnetic fluctuations were removed from the simulation indicating the electromagnetic nature of the issue. The simulations that do saturate have predicted fluxes above the available power from the heating indicating that this scenario did not have sufficient heating power consistent with the turbulent transport. Furthermore, optimization is required when designing the ramp-up, which necessitates a need for an accurate reduced transport model that is computationally tractable in an integrated modelling suite. To begin to address this, comparisons to the TGLF transport model [25] were made and none of the existing saturation rules was found to accurately capture the fluxes throughout the entire ramp-up. Moreover, the linear eigensolver within TGLF was not well suited to capture either the electrostatic or electromagnetic turbulent modes throughout the ramp-up due to the higher safety factor during the early phase and the higher *β* during the later phase, further motivating the need for a reduced model valid in the STEP regime.

The application of machine learning (ML) to the modelling of plasma turbulence has been a parallel focus to allow for usage in integrated modelling suites. Reduced linear solvers struggle with electromagnetic turbulence so a surrogate model using Gaussian process regression (GPR) has been developed for modelling the linear properties of MTMs across a seven-dimensional STEP-relevant parameter space. This approach is suited for smaller databases as generating large databases of linear MTM simulations can still be computationally expensive. Large areas of this seven-dimensional space were found to be stable, so a two-step approach was taken where initially a classifier was trained to find the stability manifold of the MTM, while a regressor model learned the linear properties in the unstable regions. The models were trained iteratively in tandem, with the MTM hit rate increasing from 33% to 71% after a single iteration. The surrogate models were able to predict the modes’ behaviour globally along with accurately capturing transition points such as critical *β* [26]. With the success of the MTM GPR, the focus has now shifted to developing a surrogate model for the KBMs.

### (b) Plasma facing component design

**Figure 4.**
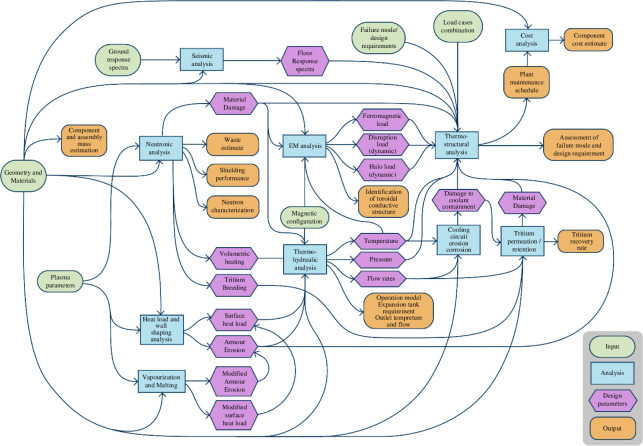
The in-vessel system workflow for analysing design concepts.

The approach to in-vessel system design has followed a similar strategy to the plasma design: the development of workflows often using lower fidelity systems models (see §5) linked together to support the ‘Decide and Iterate’ plasma facing components (PFC) design methodology described in [27]. The analysis workflow shown in figure 4 has been used to assess PFC concepts against the inputs (green) and requirements (red) from the wider plant within the ‘Design and Iterate’ PFC workflow shown in fig. 2 in [27].

In figure 4, each analysis component (blue) is supported by a set of modelling and simulation tools and methods, including both well-established and developing capabilities, and is often itself a complex workflow. An example of this is the heat loads and wall shaping analysis which, as detailed in [27], requires multiple software tools and workflows to provide the surface heat loads and armour erosion rates for any concept.

While most of the workflow is now established, there remain gaps in capability—for example, the need to model vaporization and melting effects, which remains a challenging task. Connections between analyses also need to be further developed as the design progresses—for example, at present, there is no coupling between the neutronics (neutron radiation transport) and the thermohydraulic analysis in the workflow shown in figure 4, this will however be necessary (to account for Doppler broadening—the temperature dependence of the cross-sections of neutron–nucleus interactions) as the design progresses further.

## 3. The STEP digital twin

The current STEP digital shadow for design, as outlined in the previous section, must evolve to be useful for more detailed design and eventually be combined with real-time data to form the digital twin. Although it is currently by necessity a simpler virtual representation of the plant, it is still a complex set of tools and models that is constantly iteratively improving as the understanding of the processes and interactions increases. At every stage, the gaps in our knowledge and understanding become clearer, and limitations and blockers are identified, and so a strategy is developed to address each to help move from digital shadow for design to digital twin for prediction and whole plant simulation.

Three key challenges relating to this can be identified from current experience with the STEP digital shadow:

1. Managing the complexity and quantity of information to better map out the interconnections and processes within the system. Information and knowledge management techniques such as common ontologies are critical to managing this complexity.
2. Improving the reliability, robustness and flexibility of the digital shadow to permit a move from rapid concept design iteration to more detailed design.
3. The validity and performance of the digital shadow need to be well-understood before the complexity grows. The confidence in predictions must then be traceable to underlying assessments of performance, allowing identification of when decisions are based on established science and experimental data and when they are based by necessity on extrapolation to new science and knowledge.

For the first point, digital solutions under development such as the Plant Information Management System (PIMS) will start to link information together beyond just the simulation. Similarly, the MBSE approach discussed in [5] will aid in mapping out the relations between systems. This will allow the digital shadow to move from the largely sequential workflow between systems to capturing key feedback between them and accelerating the design process. This will be underpinned through the use of advanced simulation tools to better identify key coupled physics processes, which will guide the exploration of the limited design space the STEP device occupies.

Improvements to the reliability, connectivity and flexibility of the individual data management solutions and simulation tools will similarly accelerate this process, removing unnecessary delays from each iteration, and allowing seamless pivoting between concepts. PIMS will be crucial here, as will standardization of interfaces and data formats between tools (a recognized challenge for digital twins [28]), as well as the implementation of key software engineering principles to improve the tools themselves.

Finally, the validity and performance of digital twins are a widely known challenge [28]. It is therefore vital to rigorously address these within the digital shadow and build confidence, through for example the use of uncertainty quantification methods and benchmarks. Experimental facilities such as HIVE [29], CHIMERA [30], LIBRTI [31] and MAST-U [32] will further provide a valuable test bed not just through experimental data for physical components, but of components of the digital twin as well. The initial steps to do this for parts of the larger set of coupled PFC workflows and processes are underway for CHIMERA. Similarly, digital twin development for HIVE—a facility aimed at testing high volumetric heat loads on components via an induction heating system—is also underway. These facilities will also test the data management and data pipeline aspects of the workflows, both crucial in the evolution towards the digital twin.

## 4. Supporting digital themes

The evolution of the digital shadow into the digital twin will be supported by advances across a variety of digital capabilities. These will help address the three key challenges outlined in the previous section and help drive a shift from a research regime to a delivery regime (as discussed in [5]). They can be split into nine supporting digital themes:

1. Plasma simulation—arguably one of the most critical of all our digital capabilities. The ultimate aim is a comprehensive and fully integrated STEP plasma simulation with predictive capability. At a high level, this will require (i) developing a comprehensive set of three-dimensional simulations of all the relevant physical processes in the plasma, (ii) the complex task of integrating all these together, (iii) implementing the code in such a way that enough computing power can be deployed, and (iv) enough empirical data to have confidence in the results. This is required as there is insufficient time to deliver STEP through conventional test-based development; instead, our simulation capability must be a predictive capability built upon state-of-the-art data and model hybrid simulation—suitable for taking large but measured steps around the choice of plasma state. This will require close collaboration between plasma scientists and engineers on the fusion domain side, alongside high-performance computing (HPC) specialists, ML specialists, mathematicians and data scientists across the international community. The result will be an ability to deploy a new ‘probabilistic’ approach to plasma scenario design, built upon a synthesis of models and real-world data rather than the current conservative approach based largely upon experiential data and knowledge.
2. Material simulation—this is of similar critical importance to STEP as plasma simulation. Fusion materials problems represent a large-scale computing grand challenge. Facilities such as LIBRTI [31] and IFMIF-DONES [33], although incredibly important for the fusion roadmap, will either not be able to qualify the properties of functional materials at high displacements per atom (dpa) and at plant-scale, or, not be commissioned in time to inform STEP design. This places increased emphasis on our ability to accurately simulate the effects of radiation damage in the reactor regime. The approach to materials simulation for STEP is discussed in detail in [34] and includes *in silico* mechanical testing for bulk mechanical property predictions and the Design by Fundamentals mesoscale modelling approach to crystal plasticity models.
3. Advanced engineering simulation—engineering simulation must sit alongside plasma and materials simulation in the digital shadow. As already highlighted in previous sections, the STEP device will be an incredibly complex, strongly coupled ‘system of systems’, with a multitude of physical processes to be considered, such as structural forces, electromagnetism, radiation, heat transport through solids and fluids via thermal hydraulics, and chemical transport. Advanced engineering simulation provides the capability to simulate these systems accurately at the necessary fidelity.
4. Data and digital thread—alongside simulation, data are underpinning capability that delivers the ability to predict; both are of critical importance to a successful STEP programme. This includes exploitation of the latest advances in artificial intelligence (AI)/ML and high-performance data analytics (HPDA), for example for constructing data-driven ‘foundation models’ built around multi-modal data sources.
  STEP must work with its partners and the fusion supply chain to ensure collaboration between different organizations and tools alongside a meta-data-rich Digital Thread, as discussed in [5]. Capturing the vast amounts of data generated during the programme is an ongoing task, and has seen the development of a number of bespoke systems, such as the Concepts Database—a database with parameters relating to the concepts being considered; the Providence database for data relevant to the tritium fuel cycle modelling [8], as well as various system-specific design and configuration databases (such as that described by [7] for the magnets data-centric design process). PIMS will consolidate these separate systems, and provide the digital enablement needed to underpin the information baseline of STEP [5].
5. Advanced control—covering more than just plasma control, this is essential for ensuring that the world’s first prototype commercial powerplants are robust and resilient and that new fusion facilities such as STEP achieve their design goals. This is achieved by embedding controllability in the plant design itself (rather than simply retrofitting control policies and systems) and through the deployment of advanced dynamic control (e.g. of the thermonuclear plasma). The STEP control approach is discussed in [9].
6. Industrial plant security—with an increasing emphasis on using digital solutions to design and realize commercial class fusion plants comes the risk of cyber-attack. Breaches in IT and operational technology (OT) security can also have severe consequences for a digitally controlled plant. It is essential therefore that STEP, working closely with UKAEA and its other partners, enhance cybersecurity for industrial automation and control systems (IACS)—aligned to HSE OG 86 and IEC 62443 standards. If we are to safely exploit UKRI’s fleet of exascale class supercomputers [35], STEP’s cybersecurity must stretch beyond its own sites and facilities— security must be embedded across the entire ecosystem.
7. Regulation—the use of digital technologies will need to conform to existing regulatory requirements for industry as well as emergent requirements for fusion [36]. See [6] for a discussion of regulation in relation to control for STEP. Digital technologies will also enable the discharge of legal and regulatory obligations, for example, the implementation of safety through the safety instrumented systems functional safety lifecycle standards and Secure-by-Design (SbD) for cybersecurity, or by ensuring complete traceability of data and decisions.
8. Digital technologies—digital technologies such as AI/ML, HPC, global connectivity, information sharing, and digital services have revolutionized a huge range of sectors. All of these will be crucial in tackling the simulation and data challenges required to deliver commercial fusion energy.
9. Digital disciplines—essentially the people and processes within the digital fields. Exploiting the world’s most powerful supercomputers and state-of-the-art algorithms for delivering AI solutions requires world-leading software engineering expertise and capacity matched to programme aspirations. It is critical that digital skills are developed alongside digital technologies.

Significant effort has been directed across all nine digital themes listed above, although more so in some areas than others due to the current stage in the lifecycle, and for some areas has been covered within other articles in this issue. It should be noted too that advances will not and are not being realized by STEP alone, but rather via partnerships, international collaborations, and an expanding international digital supply chain.

The following sections discuss the progress on the advanced simulation (themes 1–3), digital technologies and digital disciplines themes in further detail.

### (a) Advanced simulation

Modelling and simulation fundamentally underpin the digital shadow and the digital twin—with the ability to simulate complex plasma dynamics, magnetohydrodynamics, and engineering phenomena, whole plant modelling has the potential to accelerate the development of practical fusion energy systems. By leveraging the Denotation, Exemplification, Keying, Imputation (DEKI) principles [37], along with surrogate reasoning (where scientific representations facilitate hypothesis generation about target systems) [38] and comprehensive model representation (where the context of use informs model building and influences our perception of model efficacy) [2] a suitable set of interconnected, multi-scale, multi-physics models linked to real-world data can be constructed.

It is not necessarily the complexity of the model or its implementation that represents an advance in simulation, but the surrogate reasoning and comprehensive model representation that are their foundations. This starts from the partition of the larger system into manageable pieces—the systems simulation approach—to the creation of the individual models at the required fidelity and assessed accuracy. In the absence of experimental data, this can be supported through *in silico* validation or derivation—the use of simulation to validate a simpler analytical model or the creation of surrogate models from high-fidelity simulations via ML techniques. Verification, validation and uncertainty quantification (VVUQ) techniques ensure the reliability and proportionality of the models [11]. Advances in computing power (see §5*a*) mean simulations become more detailed and accurate through an increase in capacity and capability, for example, as discussed gyrokinetic modelling has shown great progress in exploiting petascale computing. In this section, we discuss the approach to advanced simulation, from systems models through to exascale multi-physics, within the STEP programme.

Systems simulation provides a route to fast-running and flexible digital twins. It relies on the use of models based on surrogate reasoning to simulate a set of systems and processes and is a key part of the current STEP digital shadow. The models used are simpler representations of the system than full-order simulations, either based on analytical approximations or surrogate models of high-order simulations. This approach is being tested on various facilities. For example, systems models and tools that make up the current STEP digital shadow are being used to create a digital twin of CHIMERA [39]—a facility that focuses on combined thermal and electromagnetic load testing of critical in-vessel components.

**Figure 5.**
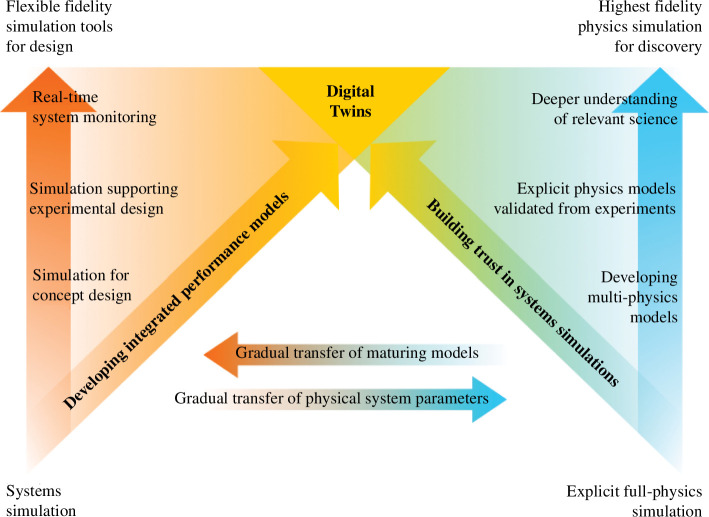
How systems simulation and explicit full-physics simulation complement each other throughout the design process in a programme such as STEP, where concept design occurs alongside physics discovery.

The use of models rather than full physics means that the systems simulation approach provides a route to a digital shadow that can rapidly evaluate many concepts in parallel. This does not, however, mean that full-physics simulation has no place: on the contrary, it is fundamental to this process in fusion, as it provides the only way to fully explore the complex physical processes in the absence of comprehensive experimental data and to develop the surrogate reasoning around them. The two approaches will remain connected throughout the future development of the STEP digital shadow and digital twins, as shown in figure 5.

High-fidelity simulation also provides the capability for *in silico* validation when validation via physical experiments is not possible. This can take many forms, from validation at the whole plant level down to validation of individual models used within workflows for sub-systems (c.f. figure 1). While the current digital shadow aims to do the former at a level of fidelity appropriate to the current design stage, *in silico* validation using higher fidelity simulation is also being used for the latter.

An example of this is the validation of a simplified model of the pressure drop for a liquid metal flowing through a rectangular, electrically conducting duct in a uniform and constant magnetic field. This liquid metal model was part of the thermohydraulic analysis (as shown in figure 4) used to assess the feasibility of liquid metal coolants for the STEP PFCs, and liquid metal breeders. This analysis informed an understanding of the impact of MHD on the breeder and coolant choice within the blanket (see the discussion in [8]) and decisions around the PFCs (see the discussion in [27]).

**Figure 6.**
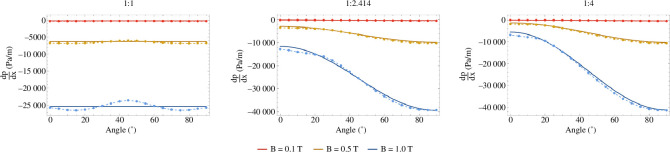
*In silico* validation of a model of the pressure drop for a liquid metal flowing through a rectangular, conducting channel in an inclined magnetic field (angles between 0° and 90°). Results from CFD simulations (dashed line and filled circles) are compared with the predictions of the model (solid line) for three different aspect ratios of the channels and three different magnetic field strengths. The model reduces to the non-inclined case at both 0° and 90°, where it is validated against experimental data.

An analytically derived model validated against experimental data is available for a constant and uniform magnetic field oriented parallel to the width or height of the pipe [40–42]. An extension to this model for the case where the field is inclined relative to the width or height of the pipe was needed, however, there is currently no experimental data available to validate this new model (and it remains an area of active research [43]). CFD simulation with Ansys Fluent was used to perform a set of numerical experiments to generate a dataset for validation of an analytical extension to the model, after first validating this approach by creating a dataset for the non-inclined case to compare to the physical experimental data. This procedure of *in silico* validation, as shown in figure 6, aids the assessment of analysis performed using the model. While in this case physical experiments could be performed, the rapid validation of the new model via simulation was key to building confidence during the rapid decision-making process in recent iterations of the PFC design.

The preceding example demonstrates the value of existing simulation tools. However, there are simulation applications within STEP that require additional capabilities that are presently not available: both the ability to rapidly prototype bespoke full-physics models to explore processes that are otherwise poorly understood as they are outside of the conditions typically seen in other applications (e.g. high magnetic fields, exceptionally steep temperature gradients, multiple processes on similar length scales that are not easily decoupled) and the ability to exploit the rapidly increasing computing power now available to run *in silico* validation of larger components and systems than currently possible.

This type of simulation is challenging for even sub-systems, and for STEP must also be done in a context requiring much higher levels of quality and assurance than typical research in this area. A good comparator is an advanced simulation for fission reactors, and the approach taken has drawn on programmes within this area, such as the ExaSMR project [44]—a US programme spun out of the US Exascale Computing Project [45] and targeting the *in silico* design and optimization of small modular reactors. This programme is transforming nuclear design in the US by integrating both new and existing nuclear reactor codes to accelerate and de-risk the design of complex engineering systems that are strongly synergistic with fusion plants.

There are two extremes in the approach for explicit full-physics simulation of tightly coupled processes—a single framework with bespoke solvers for all physics, or adaptors and interfaces between solvers from separate bespoke software libraries. The principles of software engineering would initially suggest the second as the better solution, however, it requires significant community support for interface standards. The adoption of such standards has happened in other software fields (see the discussion in §5*b*) and there was a drive for it within the Exascale Compute Project with xSDK—the Extreme-scale Scientific Software Development Kit [46]. This allows common linear and nonlinear solvers to be called via a common interface, already used within the MOOSE framework [47] via its PETSc interface. The requirements of such an approach necessitate consideration of how the coupling is done between two potentially vastly different solution approaches as well as issues surrounding the transfer of data between those spaces in an accurate manner.

The strategy for STEP has thus been to use a pragmatic compromise of the two approaches, by developing a suite of applications within MOOSE that make use of the wide array of validated functionality within the framework alongside complementary libraries. This has included: Apollo, an electromagnetics solver that can solve both directly using MOOSE and with the MFEM library [48]; AURORA, a coupling of OpenMC with MOOSE for coupled nuclear analysis [49]; and Hippo, a prototype coupling of OpenFOAM with MOOSE. Similar compromise approaches have also been undertaken for advanced fission reactor design [50].

The selection of a common framework was based on an assessment against key requirements: software and model quality, architecture design for scalability, portability to a variety of HPC hardware architectures, flexibility and modifiability and support and documentation. MOOSE was selected after a detailed consideration of 36 candidate frameworks against these requirements, primarily due to its high software quality (as it is used in the design of advanced fission reactors and so must meet stringent quality requirements) and its scalability.

Challenges have been encountered with the toolset development, largely relating to the inherent compromise nature of the solution: in particular it has proved difficult to couple some libraries and solvers to the framework. This has typically happened when the application to be coupled in does not have a suitable application programming interface (API) or architecture to allow in-memory transfer of data (and thus avoid the significant performance issues with the alternative of a read/write to disk at every timestep).

The adoption of the MOOSE framework has, however, benefitted a number of areas within the programme, for example, the Design by Fundamentals materials models discussed in [34] are being implemented within the framework as it provides a far more scalable solution that will be needed in the near future for more detailed component design. Another example is the implementation of custom algorithms for the mapping of electromagnetic forces on dissimilar finite element meshes, resolving issues encountered with this in existing tools [51]. A combined MHD and CFD implementation for simulations of the flow of liquid metals in high magnetic fields is also in progress and will permit more detailed analysis which builds on the work presented above. The digital twin development for HIVE is making use of Apollo—an initial validation workflow for a simplified monoblock is being built here, to simulate the coupled electromagnetic and thermomechanical processes. Application of the wider toolset is now starting to move towards simulations of whole systems, such as breeder blankets [52], divertors and magnet systems.

Alignment with international programmes is critical, as these are complex codes requiring significant development. There has been collaboration here in a number of areas, for example on the Cardinal application, a coupling of NekRS and OpenMC within the MOOSE framework [53]. Another key example is the international effort directed at improvements to DAGMC [54] and OpenMC [55] that STEP has supported. These together now provide an excellent capability for high-fidelity neutronic simulation for fusion applications, and both the Apollo and Cardinal applications will support more detailed analysis and simulation in workflows, such as shown in figure 4, where currently only limited coupling between neutronics and other physics is considered. The improvements to OpenMC itself are already supporting stand-alone neutronic analysis within the programme.

### (b) Digital technologies

The rapid pace of development of digital technologies such as HPC, AI/ML methods and big data have been revolutionary across a huge range of sectors and have enabled leaps in tackling key outstanding challenges required to deliver commercial fusion energy.

**Figure 7.**
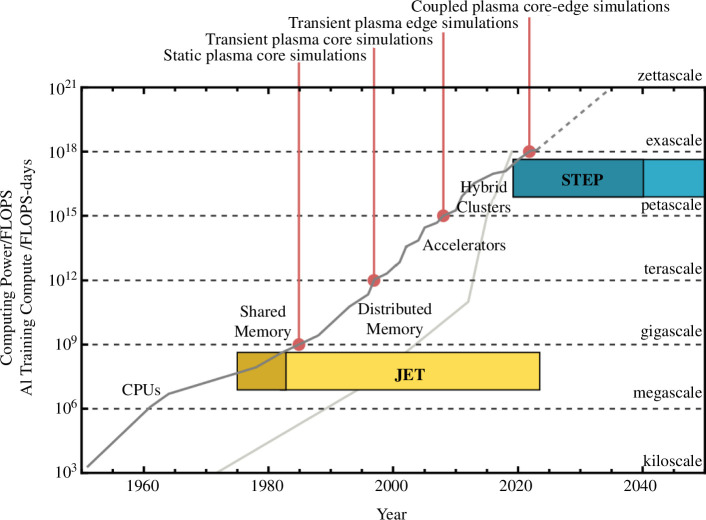
Developments in digital technologies between JET and STEP. The increase in (measured) compute power of the fastest machine or facility at the time in floating point operations per second (FLOPS) is shown as a dark grey line, and the increase in AI training compute in neural net operations per second × days (FLOPS—days) is shown as the light grey line. The timescales for the design (dark band) and operation (light band) of JET and STEP are both illustrated. Compute power data primarily from the Top500 [56], AI compute data from OpenAI [57]. Plasma simulation capabilities at different scales following [58].

Figure 7 shows the exponential growth in computing power and changing HPC paradigms that have occurred between the design of JET and the STEP programme: a growth of nine orders of magnitude in raw computing power, allowing us to move from low-fidelity static models of just the core plasma to high-fidelity transient models of the core and edge plasma. The current move to the exascale will subsequently enable high-fidelity coupled simulations of these [59] and simulations of the edge plasma interactions with the first wall [60]. Both are critical to the development of commercial fusion.

Alongside the growth of HPC has been the rapid development of AI/ML, partly enabled by the trends in HPC computing shown in figure 7—for example, the impact of the introduction of GPU acceleration on the use of computing in training AI models can be clearly seen. Exascale computing will enable the realization of AI/ML techniques for active plasma control [61,62], which will be key to the successful operation of commercial fusion plants. Alongside this, ML surrogate models will also drastically speed up integrated simulation workflows and digital design, making rapid iteration of designs possible. For example, a neural network surrogate of the turbulent transport simulation in an integrated plasma model can provide a 100-fold speed up [63]. This capability will be critical to STEP’s design, which will be largely dominated by simulations. It will also enable real-time control strategies [64,65].

Exploiting the advances in raw computing power is however itself a challenge: as processing power has increased memory bandwidth has not kept pace, and new algorithms and methods must be developed to bridge this gap [59]. The hybrid nature of new clusters requires new software engineering paradigms to create portable codes [60].

It is important to note that while there are simulation challenges that will require the most advanced exascale computing facilities, there is, as discussed in the previous section, a hierarchy of simulations: a few will require exascale to run a single simulation, but there are others that will only need the petascale and even more so below that. The challenge at each level is in the number of runs that are possible, which in turn permits exploration of the design space and the development of surrogate models. It should also be noted that AI/ML for fusion not only requires the use of computing for training (as shown by the light grey line in figure 7), but also requires it for the generation of datasets from simulation. The extensive design spaces can require codes to be run many hundreds of millions of times [63]. It is here that we can almost immediately exploit the growth in computing power—these codes require little further development, merely the facilities on which to run them to provide immediate benefits to the programme.

The use of AI/ML also offers the potential to transform Knowledge and Information Management (KIM). The use of techniques such as LLMs for KIM for programmes such as STEP will, however, require care to ensure traceability of information provided back to the source material and to avoid issues with ‘hallucinations’ (incorrect or nonsensical output), for example, by using retrieval-augmented generation (RAG) methods [66].

### (c) Digital disciplines

Recent digital capability developments are not limited to technologies. The last decade has seen the development of the research software engineering discipline [67,68]. Research software engineers bridge the gap between professional software engineering and scientific research—they are able to apply best practice from industry while understanding the research domain. This drives an iterative approach to the development of software through a lifecycle that increases the technology readiness level (TRL) alongside the development of research [68]. The application of research software engineering has driven advances in many disciplines (see e.g. [69]) and allows the exploitation of the world’s most powerful supercomputers alongside state-of-the-art algorithms. The continued development of research software engineering and other research technical professional expertise within the STEP programme will be key to exploiting enhanced computing capability and providing enhanced complex research software to the reliability, quality and integrity standards needed for the realization of commercial fusion.

Simulation software within STEP is a vast ecosystem of diverse and varied tools, from bespoke prototype scientific research code through to standard commercial off-the-shelf (COTS) tools widely used across other industries. The solution architecture—the overall architecture of the various simulation activities and the digital systems supporting them—is complex but key to ensuring a rapid pace of work on the programme. It captures vital dependencies and links between areas, as well as the movement of data and information, and will form the basis of the workflows that will make up specific digital shadows and eventually digital twins.

Therefore, a key challenge is collaboration and integration—both between software tools and between people within the programme. While it is now widely accepted in engineering that common standards for data and model exchange are fundamental to complex workflows (e.g. [70,71]), this idea is still evolving for plasma simulation software, and as is common for any prototype software, changes rapidly occur in the data formats used. However, an unexpected difference in input formats can at best disrupt workflows, causing delays while time is taken to develop new readers for the new data format, and can at worst introduce subtle errors that are only discovered later in the simulation workflow. Formally defining standards, as has been done for ITER with the ITER Physics Data Model (PDM) and Integrated Modelling and Analysis Suite (IMAS) [72], and ensuring software complies with these contracts between interfaces will benefit both the quality and performance of the simulation workflows. A wide research software engineering community within the programme will help with this challenge for software under rapid development—for example, enforcing expected interfaces and standards via automated testing (continuous integration and continuous deployment) and SecDevOps platforms.

Simulation software is by its nature numerical method focused, which brings further challenges to software engineering, especially for software used for operational plant design, which necessitates higher quality standards than for research. While software engineering best practices such as version control, automated testing and SecDevOps are now common across the research software engineering community and are being implemented in software used across the programme, this best practice is by and large not tailored to the fairly unique types of software needed for fusion. Modern software development has been dominated by the drive towards agile methods, a key feature of which is the focus away from comprehensive software documentation in favour of working software [73]. This is often incorrectly interpreted as a push away from a focus on traceability. However, this is key to the quality of scientific software— where the science behind the derivation of numerical methods and algorithms cannot be simply decoupled from their implementation. It is not practical to include rigorous derivation of models and discussions on numerical accuracy in code comments, but the code must have appropriate supporting documentation to allow users to make informed decisions on the appropriateness of a tool for their use case and for developers to understand the context behind the implementation. New methodologies and digital tools must be developed to address this gap to prevent increasing technical debt within the fusion simulation software ecosystem.

## 5. Conclusion

STEP represents a significant extrapolation from current fusion devices, pioneering the design of a future fusion power plant characterized by a scarcity of physical evidence to mitigate inherent uncertainties. Fusion power plants are intricate systems, comprising a complex web of interconnected components, with our understanding of these relationships still in its infancy due to the nascent nature of the science and technology involved. This presents a diverse array of challenges, with digital technology playing a central role in conceiving, testing and validating new ideas. The paper has demonstrated promising glimpses in plasma transport and design workflows during this conceptual design phase (Tranche 1), leveraging digital tools to work smarter and reduce the need for physical experimentation.

Given the inherent uncertainty of unprecedented systems, extensive experimentation, both physical and digital through modelling, is essential to develop viable solutions while navigating between fictional and structuralist perspectives. The digital ecosystem design must be inclusive to remove the barriers that create undue effort and separation (friction), enabling delivery partners to participate equally, confidently and independently in everyday activities. Advanced digital tools enable iterative prototyping, providing a unique capability for predicting performance, cost, schedule and risk.

These themes are integrated into the STEP programme’s vision to accelerate delivery via the development of a digital twin. The context of use guides our model-building efforts and influences perceptions of model efficacy. In developing an unprecedented fusion power plant—one that significantly extrapolates beyond existing and near-term machines—allocating HPC resources becomes crucial. Rather than solely focusing on high-fidelity predictions, the emphasis should be on surrogative reasoning [38]. This approach, using functionally representative models to generate ideas, fosters new thinking, incorporates surrogate data and employs intelligent approximations to guide decision-making. By strategically allocating computational efforts, we can advance progress while respecting resource limitations. Balancing fidelity and efficiency ensure that our technology exploitation plan maximizes impact, even within the constraints of limited resources.

Delivering this overall digital vision for STEP will rely on leveraging the most recent advances across the entire digital space. The programme must then begin to move beyond just adopting the current state of the art to defining the new digital paradigms required to realize commercial fusion.

## Data Availability

This article has no additional data.
